# Mammary Abscess in Infants

**Published:** 1890-12

**Authors:** 


### Selections and Abstracts

### MAMMARY ABSCESS IN INFANTS.

After relating the history of a fatal case of pyæmia following mammary abscess in an infant three weeks old, Dr. Frank C. Bressler, of Baltimore, concludes as follows :

"The point of most importance is the propriety or justification of indiscriminately squeezing every breast that appears swollen at birth. It seems to be the delight of the midwife, nurse, or some old, neighborly women to examine the breasts immediately, with the result of giving them the necessary squeeze and thus triumphantly exhibit a little milk, which naturally is found, on hard squeezing, in almost all infants' breasts. If the squeezing only ended here—but no ; next day the poor little victim has to go through the same process. If fortune is favorable, no evil results ; if otherwise, evil follows as above narrated. This is a pernicious practice and ought to be stopped, since an organ so tender as the breast can certainly not be so rudely handled. As a result of this practice I tell the one who is going to wash the baby that the breasts must not be touched until I have seen them. If any squeezing is to be done, I will do it, and I insist on my advice being followed. I think this a point of great interest, and, teaching the mothers the dangers that may follow such a practice, many infants' lives can be saved that are otherwise made unhappy by the pain, later on by deformity of the breast, besides the possible loss of life."—*Archives of Pediatrics.*

